# Workforce preparedness for disasters: perceptions of clinical and non-clinical staff at the U.S. Department of Veterans Affairs

**DOI:** 10.1186/s12889-020-09597-2

**Published:** 2020-10-02

**Authors:** Aram Dobalian, Michelle D. Balut, Claudia Der-Martirosian

**Affiliations:** 1 Veterans Emergency Management Evaluation Center, U.S. Department of Veterans Affairs, 16111 Plummer St. MS-152, North Hills, CA 91343 USA; 2 Division of Health Systems Management and Policy, University of Memphis School of Public Health, Memphis, TN USA

**Keywords:** Clinicians, Non-clinicians, Workforce preparedness, Natural disasters, Epidemics, Pandemics, United States Department of Veterans Affairs, Disasters, Emergency preparedness

## Abstract

**Background:**

Most U.S. studies on workforce preparedness have a narrow scope, focusing primarily on perceptions of clinical staff in a single hospital and for one type of disaster. In contrast, this study compares the perceptions of workplace disaster preparedness among both clinical and non-clinical staff at all U.S. Department of Veterans Affairs (VA) medical facilities nationwide for three types of disasters (natural, epidemic/pandemic, and manmade).

**Methods:**

The VA Preparedness Survey used a stratified simple random, web-based survey (fielded from October through December 2018) of all employees at VA medical facilities. We conducted bivariate and multivariate logistic regression analyses to compare the sociodemographic characteristics and perceptions of disaster preparedness between clinical and non-clinical VA staff.

**Results:**

The study population included 4026 VA employees (2488 clinicians and 1538 non-clinicians). Overall, VA staff were less confident in their medical facility’s ability to respond to epidemic/pandemics and manmade disasters. Depending on the type of disaster, clinical staff, compared to non-clinical staff, were less likely to be confident in their VA medical facility’s ability to respond to natural disasters (OR:0.78, 95% CI:0.67–0.93, *p* < 0.01), pandemics (OR:0.82, 95% CI:0.70–0.96, *p* < 0.05), and manmade disasters (OR: 0.74, 95% CI: 0.63–0.86, *p* < 0.001). On the other hand, clinicians, compared to non-clinicians, were 1.45 to 1.78 more likely to perceive their role in disaster response to be important (natural OR:1.57, 95% CI:1.32–1.87; pandemic OR:1.78, 95% CI:1.51–2.10; manmade: OR:1.45; 95% CI: 1.23–1.71; *p*’s < 0.001), and 1.27 to 1.29 more likely to want additional trainings to prepare for all three types of disasters (natural OR:1.29, 95% CI:1.10–1.51; pandemic OR:1.27, 95% CI:1.08–1.49; manmade OR:1.29; 95% CI:1.09–1.52; *p*’s < 0.01). Clinicians were more likely to be women, younger, and more educated (*p*’s < 0.001) than non-clinicians. Compared to clinicians, non-clinical staff had been employed longer with the VA (*p* < 0.025) and were more likely to have served in the U.S. Armed Forces (*p* < 0.001).

**Conclusions:**

These findings suggest both a desire and a need for additional training, particularly for clinicians, and with a focus on epidemics/pandemics and manmade disasters. Training programs should underscore the importance of non-clinical roles when responding to disasters.

## Background

Many healthcare workers, including those who directly or indirectly deliver care and services to patients [1], report that they often feel unprepared to effectively respond to major disasters [2–10]. Some of the concerns they express include: transportation problems, safety of self and family members, caretaking obligations, personal health issues, lack of personal preparedness, lack of confidence in medical facility’s ability to respond effectively, insufficient training, and unwillingness to report to work [11–19]. The type of disaster may also be a potential barrier for healthcare workers to report to work. Some studies have found that healthcare workers are more willing to respond to natural disasters and mass casualty events, but less likely to respond to infectious outbreaks or epidemics and radiological or chemical events [12–14, 20–22]. Most U.S. studies on workforce preparedness, however, have a narrow scope, focusing on perceptions of clinical staff in a single hospital. Clinicians and non-clinicians both have important roles to play during disasters. Nevertheless, the education and professional socialization of clinicians and non-clinicians differs, and thus it is important to understand whether preparedness differs between these groups. Furthermore, most existing studies examine one type of a disaster event. These limitations may hamper efforts to effectively plan for different types of hazards.

After the Department of Defense, the U.S. Department of Veterans Affairs (VA) is the second largest U.S. federal government agency, with more than 360,000 employees. The VA is also the largest integrated healthcare system in the U.S., providing care to more than 9 million Veterans at 1255 healthcare facilities, including 170 VA Medical Centers and 1074 outpatient sites. Understanding the perceptions of preparedness at the workplace for all VA healthcare employees, including both clinical and non-clinical staff, is critical to ensure the continued delivery of timely, high-quality care to U.S. Military Veterans during and immediately after different types of disasters.

Consequently, the Veterans Emergency Management Evaluation Center (VEMEC) developed and conducted the first survey of disaster preparedness of VA healthcare employees nationwide at the U.S. The VA All Employee Disaster Preparedness Survey (VA Preparedness Survey) focused on several key factors within the context of three different types of major disasters: natural, epidemic, and manmade. The main objectives of this study were to:

- Examine VA healthcare workers’ perceptions of institutional preparedness (i.e. how prepared their facility is to respond to a disaster), the need for additional trainings, and ability to respond during the aforementioned types of disasters; and
- Understand how perceptions of workforce preparedness (i.e. how prepared is their facility’s workforce to respond to a disaster) might differ between clinical and non-clinical staff.

## Methods

### Study design

The VA Preparedness Survey was a quantitative, quality improvement [23] study designed to gather information from VA employees about their preparedness at work. Sociodemographic data collected by the survey included: age, gender, education, military service, ethnicity, race, marital status, household composition (dependents under the age of 17), pet ownership, and information on employment (full-time or part-time, length of employment at the VA, supervisory status, and clinical cf. non-clinical responsibilities). The study questions regarding perceptions of workforce preparedness included: (a) perceptions about institutional readiness; (b) desire for additional training; (c) understanding of their role during disaster response; and (d) perceptions about the importance of their role during disasters (see VHA Preparedness Study Questionnaire).

The study defined a major disaster as one that causes disruption in services, mass injury, loss of life, or widespread damage to property, and requires resources outside of the local community to help with recovery efforts. More specifically, the three types of disasters included in the survey were defined as: (1) a natural disaster (an earthquake, hurricane, tornado, flood, wildfire, or severe winter storm); (2) an epidemic (a widespread infectious disease such as a pandemic influenza); and (3) a manmade disaster (a mass shooting, leak from a nuclear power plant, or a dirty bomb).

### Questionnaire

The VA Preparedness Survey was a random, anonymous, 10-min web-based survey that was fielded from October through December 2018. Invitations to participate in the survey, as well as four reminders, were sent to the official VA email addresses of potential study participants. The emails provided the website URL address of the survey. The web survey was developed to be a single page, scrolling survey, and was designed and formatted to support ease of navigation and limit mode effect errors. The survey’s design and implementation are consistent with generally accepted recommendations for such studies [24]. The survey questions regarding disaster preparedness were measured using a five-point Likert scale (5 = strongly agree, 4 = agree, 3 = neutral, 2 = disagree, 1 = strongly disagree) and they were all asked within the context of each disaster type (natural, epidemic, or manmade). The survey instrument used several validated scales from various sources, all of which are freely available online, including: The Behavioral Risk Factor Surveillance System Questionnaire from the U.S. Centers for Disease Control and Prevention [25], which was used to inquire about household preparedness; the American Community Survey from the U.S. Census Bureau [26], which was used to capture demographic data from employees, and; the All Employee Survey from the VA [27], which was used to capture VA employment characteristics and veteran status.

### Study population

The target population of the survey consisted of all full-time and part-time employees at U.S. VA medical facilities nationwide. The sampling frame was assembled by processing the most current email data set of all employees at VA medical healthcare centers, excluding VA headquarters (central office) staff, residents, students, work study members, trainees, fellows, contractors, interns, volunteers, and those with incomplete administrative information. The sampling frame consisted of 362,823 VA employees at medical facilities, and a stratified simple random sample of 25,000 was selected, 6250 from each of the four U.S. regions (north, south, east, west). Both clinical and non-clinical staff were invited to participate. Clinical staff were defined as spending at least 20% of their work time performing clinical duties (outpatient care, inpatient care, or extended care). Non-clinical staff were defined as those working in administration, education, research, and affiliated areas. A total of 4026 completed the web-based survey with an adjusted response rate of 20%.

### Statistical analyses

Bivariate analyses were used to compare the demographic characteristics of clinicians and non-clinicians. Chi-square tests were used to assess the differences between clinical and non-clinical staff’s perceptions of institutional preparedness during three types of major disasters. Each dependent variable was dichomotized by combining the “strongly agree/agree” responses into one category and the remaining responses (“strongly disagree”, “disagree”, and “neutral”) into another category. To further compare perceptions of four workforce preparedness for the three types of disasters (natural, pandemic, man-made), 12 multivariate logistic regression analyses were conducted, one for each dependent variable, while controlling for relevant study covariates such as: age (18–44, 45–54, 55–64, 65+), gender (male vs. female), race (white vs. non-white), Hispanic (yes/no), married (vs. not currently married), have dependents less than 17 years of age (yes/no), having pets or service animals at home (yes/no), length of employment at the VA (< 1 year, 1–3 years, 4–5 years, 6–10 years, or 10+ years), served in the US Armed Services (yes/no), and having supervisory responsibilities at work (yes/no). The selection of which covariates to include in the regression analyses were guided by both statistical significance and theory. Additionally, to account for the survey design weights, the svy command in Stata/SE (Version 15, StataCorp LP, College Station, Texas USA) was used when conducting the logistics regression analyses. Odds ratios and 95% confidence intervals (95% CI) for clinical and non-clinical comparisons were calculated for the perceptions of workforce preparedness dependent variables. Statistical significance was set at *p* < 0.05. The survey data were analyzed using STATA/SE software. The reliability and validity of survey responses for workforce preparedness and identified associated factors were assessed and confirmed by conducting several sets of analyses, such as Pearson’s r and Spearman’s rank correlations, regression, and principal component analyses, using the full Likert scale as well as the dichotomized responses. The data were weighted using post-stratification weighting procedures to represent national-level population(s) of all VA employees at medical facilities. The results presented below are weighted to the national level.

## Results

### Characteristics of participants

Table 1 lists the sociodemographic characteristics of clinical and non-clinical staff. Of the 4026 employees who responded to the survey, 2488 (62.2%) were identified as clinical staff and the remaining 1538 (37.8%) were identified as non-clinical staff. Gender, age, education, military status, and length of time at VA were the only sociodemographic characteristics that showed significant differences between clinical and non-clinical staff. The results indicate that, compared to clinicians, non-clinicians were more likely to be men (68.2% vs. 57%, *p* < 0.0001), slightly older (45 and older: 76.1% vs. 69.1%, *p* < 0.0001), less likely to have a graduate degree (26.8% vs. 39.1%, *p* < 0.0001), more likely to be employed at the VA for a longer period of time (10+ years: 40% vs. 35.5%, *p* < 0.05), and more likely to have served in the U.S. Armed Forces (47.4% vs. 26.8%, *p* < 0.0001).

**Table 1:** Socio-Demographic Characteristics of VA Employee, Clinical vs. non-Clinical Staff

	Clinical Staff^a^ (*n* = 2488, 62.2%)	Non-Clinical Staff^a^ (*n* = 1538, 37.8%)	Chi_Square	***p***-value
Male	31.8%	43.0%	35.94***	0.000
Age Categories:				
18–24	0.6%	0.6%		
25–34	10.1%	6.1%		
35–44	20.2%	17.3%		
45–54	28.6%	34.9%		
55–64	33.2%	34.5%		
65+	7.3%	6.7%	5.99***	0.000
Spanish, Hispanic, or Latino	8.7%	10.0%	1.45	0.228
White	70.0%	68.5%	0.89	0.346
Married or living with a partner	70.4%	68.5%	1.35	0.246
Has pets or service animals	60.9%	59.3%	2.40	0.122
Has dependents 17 years or younger	34.6%	31.5%	3.63	0.057
Education:				
No college	10.8%	12.9%		
Some college or Associate degree	26.1%	34.1%		
Bachelor’s degree	24.1%	26.2%		
Graduate degree	39.1%	26.8%	19.51***	0.000
Served in the US Armed Forces	26.8%	47.4%	146.99***	0.000
Length at the VA (in years):				
Less than 1 to 3	31.2%	28.3%		
4 to 10	33.4%	31.7%		
10 or more	35.5%	40.0%	3.67*	0.025
Has supervisory responsibility	29.9%	31.4%	0.86	0.323

Note: Chi-square tests were conducted to make comparisons between clinical and non-clinical staff

*p < 0.05, **p < 0.01, ***p < 0.001

^a^Weighted percent

### Perceptions of disaster preparedness

Table 2 compares the perceptions of disaster preparedness between VA clinical and non-clinical staff. VA clinical staff were less confident in their VA medical facility’s ability to respond to a natural disaster (60.2% vs. 65.0%, *p* < 0.05), an epidemic (53.1% vs. 57.1%, *p* < 0.01), and a manmade disaster (47.5% vs. 54.1%, *p* < 0.01), compared to non-clinical staff. Compared to non-clinical staff, clinical staff were more likely to want additional trainings for a natural disaster (62.5 vs. 57.2, *p* < 0.01), an epidemic (65.5 vs. 59.8, *p* < 0.01), and a manmade disaster (70% vs. 63.8, *p* < 0.01). The findings also indicate that VA clinical staff were more likely to consider their role to be important to their medical facility’s response to disasters, whether natural disasters (75.2% vs. 676%, *p* < 0.001), epidemics (72.3% vs. 61.6, *p* < 0.001), or manmade disasters (72% vs. 63.7%, *p* < 0.001). There was no statistically significant difference between clinicians and non-clinicians in terms of their understanding of their role during disaster response.

**Table 2:** Perceptions of Workforce Preparedness during Disasters, Clinical vs. non-Clinical Staff

	Clinical Staff^a^	Non-Clinical Staff^a^	Chi-Square	***p***-value
Confident in my facility’s ability to respond to a(n)^b^:				
Natural disaster	60.2%	65.0%	4.43*	0.012
Epidemic	53.1%	57.1%	5.91**	0.003
Manmade disaster	47.5%	54.1%	7.19**	0.001
Would like additional training to prepare for a(n)^b^:				
Natural disaster	62.5%	57.2%	5.06**	0.006
Epidemic	65.5%	59.8%	6.02**	0.002
Manmade disaster	70.0%	63.8%	7.67**	0.001
Understand my role in my facility’s overall response to a(n)^b^:				
Natural Disaster	57.0%	57.5%	1.16	0.312
Epidemic	49.1%	48.2%	0.12	0.891
Manmade Disaster	48.6%	51.2%	1.17	0.312
My role in my facility’s overall response is important during a(n)^b^:				
Natural Disaster	75.2%	67.6%	12.80***	0.000
Role in Epidemic	72.3%	61.1%	25.78***	0.000
Manmade Disaster	72.0%	63.7%	13.23***	0.000

Note: Chi-square tests were conducted to make comparisons between clinical and non-clinical staff

*p < 0.05, **p < 0.01, ***p < 0.001

^a^Weighted percent

^b^Responded “strongly agree” or “agree”

Table 3 presents the odds ratio and 95% Confidence Interval (95% CI) for workforce preparedness comparing clinical and non-clinical staff after controlling for study relevant variables such as: age, gender, race, Hispanic, marital status, having pets or service animals, having dependents less than 17 years of age, education, served in the US Military, length of employment at the VA, and having supervisory responsibilities. The results from these multivariate analyses confirm the findings from the bivariate analyses and indicate that clinical staff, compared to non-clinical staff, were less likely to be confident in their VA medical facility’s ability to respond to natural disasters (OR:0.78, 95% CI:0.67–0.93, *p* < 0.01), pandemics (OR:0.82, 95% CI:0.70–0.96, *p* < 0.05), and manmade disasters (OR: 0.74, 95% CI: 0.63–0.86, *p* < 0.001). On the other hand, clinicians, compared to non-clinicians, were more likely to perceive their role in disaster response to be important for natural disasters (OR:1.57, 95% CI:1.32–1.87, *p* < 0.001), pandemics (OR:1.78, 95% CI:1.5.10–2.10, *p* < 0.001), and manmade disasters (OR:1.45, 95% CI:1.23–1.71, *p* < 0.001). Similarly, compared to non-clinicians, clinicians were more likely to want additional training to prepare for all three types of disasters (natural disasters OR:1.29, 95% CI:1.10–1.51, pandemics OR:1.27, 95% CI:1.08–1.49; manmade disasters OR:1.29, 95% CI:1.09–1.52; *p*’s < 0.01). With regard to understanding their roles during disaster response, there were no differences between clinical and non-clinical staff (see Table 3).

**Table 3:** Odds Ratio^a^ for Perceptions of Workforce Preparedness^b^ during Disasters, Clinical vs. non-Clinical Staff

	Odds Ratio	95% Confidence Interval (95% CI)	***p***-value
Clinical staff less confident in their facility’s ability to respond to a:			
Natural disaster	0.78**	0.67, 0.93	0.004
Pandemic/Epidemic	0.82*	0.70, 0.96	0.012
Manmade disaster	0.74***	0.63, 0.86	0.000
Clinical staff would like additional training to prepare for a:			
Natural disaster	1.29**	1.10, 1.51	0.002
Pandemic/Epidemic	1.27**	1.08, 1.49	0.004
Manmade disaster	1.29**	1.09, 1.52	0.003
No difference between clinical and non-clinical staff in terms of understanding their role in their facility’s overall response to a:			
Natural Disaster	1.03	0.88, 1.21	0.676
Pandemic/Epidemic	1.08	0.92, 1.26	0.345
Manmade Disaster	0.96	0.82, 1.12	0.601
Clinical staff more likely to agree their role in facility’s overall response is important during a:			
Natural Disaster	1.57***	1.32, 1.87	0.000
Pandemic/Epidemic	1.78***	1.51, 2.10	0.000
Manmade Disaster	1.45***	1.23, 1.71	0.000

^a^Twelve separate logistic regressions were conducted. For each logistic regression, the following covariates were included: gender, age, race, Hispanic, married, having pets or service animals, having dependents less than 17 years of age, education, served in the US Armed Forces, length of employment at the VA, and having supervisory responsibilities

^b^Responded “strongly agree” or “agree”

*p < 0.05; **p < 0.01; ***p < 0.001

## Discussion

As the largest integrated healthcare system in the U.S., the VA plays an important role in the nation’s disaster preparedness and response [28]. In fact, one of VA’s healthcare missions is to provide backup medical resources to both the military health system and to local communities following terrorist incidents and other major disasters [29]. As such, VA has responded to numerous national emergencies [30] and also provided care for non-Veterans during such events [30–33]. Nevertheless, we found that 57–70% of VA employees would like more training to improve their level of preparedness for a disaster, with the highest proportion of employees reporting the need for training related to epidemics and manmade disasters compared to natural disasters. These findings are consistent with other studies which found that healthcare workers are more likely to be willing and able to respond to natural disasters and less likely to be willing and able during infectious outbreaks and radiological or chemical events [12–14, 16, 21].

Other studies have shown that healthcare workers with a specified role (i.e. a specifically defined role for the individual during a disaster) were three to five times more likely to respond during a disaster than those without a specified role [2], and those with a higher perceived importance of their role in an emergency were also more able and willing to report to work during a disaster [15, 16]. Studies have also found that non-clinical healthcare workers were significantly less willing to respond during catastrophic events than their clinical counterparts [2, 13, 21, 22, 34]. Since only around three in five non-clinicians considered their role in disaster response to be important compared to a quarter of clinicians, training programs should target non-clinical staff and emphasize their importance when responding to a disaster.

Perceptions of physical safety and confidence in their facility’s ability to handle and respond to a major disaster have also been reported to influence healthcare workers’ willingness and ability to respond [8, 17]. Prior studies have found that only one-half of respondents were confident that they would be safe at work during a natural disaster or pandemic influenza, whereas only one-third responded the same regarding a radiological event [10, 16]. Non-clinical staff were typically more confident in their hospital’s ability to provide safety precautions compared to clinical staff [35]. These findings are consistent with this study, which indicated that non-clinical staff are more confident than clinicians in their medical facility’s ability to respond to natural disasters, epidemics, and manmade disasters. It is possible that clinicians are less confident than non-clinicians are because the former generally have a better understanding of the clinical challenges and limitations of preparing for and responding to these events compared to the latter. Future studies should examine this possibility. VA employees were more confident in their facility’s ability to respond to natural disasters (around 60%) than epidemics or manmade disasters (around 50%). Whether facilities are objectively more prepared for natural disasters than epidemics and manmade disasters is beyond the scope of the survey.

Previous studies have found that veteran households, compared to nonveteran households, were more likely to have 3-day supplies of water and food, a written disaster plan, and a 3-day supply of prescription medications [36, 37]. Healthcare practitioners with military experience were also cited as being more willing to respond during a disaster than their colleagues who had not served [2]. It has been pointed out that veterans may more likely be prepared for emergency situations because of the intense military training programs for service in combat zones [35] and many have participated in disaster response efforts during their active duty military service, thus making them more familiar with hazardous material and terrorist incidents, disaster medical operations, fire safety, and other emergency processes and procedures [38]. This study found that non-clinicians were more confident in their facility’s ability to respond to major disasters and were less inclined than clinicians to request more training to prepare. These findings may be due to that fact that almost half (47.4%) of VA non-clinicians have served in the U.S. Armed Forces compared to about a quarter (26.8%) of VA clinicians, which perhaps instilled in them a culture of preparedness.

Prior work also suggests an association between healthcare professionals’ level of education and their intention to respond to disasters. For example, previous studies have found a positive association between postgraduate education and willingness to respond to a chemical, biological, or radiological (CBR) event [2] and an influenza pandemic [16]. The researchers hypothesized that those with postgraduate qualifications were better educated about agency-specific risks, use of personal protective equipment, and organizational procedures, and therefore felt more confident about their ability to manage incidents [3]. Additionally, women healthcare workers were reported to be less willing to respond during a disaster, especially during epidemics [2, 13, 21]; and younger respondents were more likely to be absent than their older colleagues [2, 16]. Women VA volunteers within the Disaster Emergency Medical Personnel System (DEMPS) program were also somewhat less ready to deploy in the event of a disaster compared to men, perhaps due to disproportionate responsibilities for caregiving [39]. It is also possible that the observed gender differences are due to differences in risk-taking behaviors between men and women [40, 41]. Of the VA employees surveyed in our study, 64% were women (68.2% clinicians 57.0% non-clinicians) and 9.2% were under the age of 34 (10.7% clinicians 6.7% non-clinicians). These results suggest the potential for absenteeism for regularly scheduled shifts in the days following a catastrophic disaster, especially among clinical staff, who tend to be women, younger, less confident in their facility’s ability to respond to disasters, and slightly more likely to have dependents aged 17 years or younger.

### Limitations

First, the survey focused on perceptions of workforce preparedness and readiness to respond during disasters but did not ask questions about an actual event. The main goal of the study was to gather information about VA preparedness nationwide, and therefore collecting information on a specific event was not possible. Second, since this survey was conducted via email and online, the survey results might underrepresent the perspectives of employees who do not use email frequently although the survey was kept open for about 4 months. Third, staff were grouped together as either clinical or non-clinical which did not allow us to differentiate perceptions among these two broad job categories. The response rate is relatively low, potentially leading to sampling bias although sociodemographic characteristics of the sample were similar to the overall VA workforce. Finally, it is possible that respondents with greater interest in the subject were more likely to complete the survey compared to those with less interest in it; this may lead to an overestimate of the desire for additional training.

The results of our study are not necessarily generalizable outside VA. However, VA healthcare workers do not differ in their training and socialization for their professions and our results are similar to those reported in prior, smaller studies. VA respondents should not differ significantly from healthcare workers in other settings with respect to their type, scope, or amount of disaster preparedness training either during their professional training or while working at VA. For example, VA requires that its facilities be accredited by The Joint Commission, which has certain requirements about preparedness. While VA is not subject to the preparedness requirements of the Centers for Medicare and Medicaid Services because it does not receive funds from Medicare and Medicaid, VA’s own requirements meet (or in some instances exceed) these standards. Requirements for training are set by professional standards boards for individual clinicians at the state level. The applicability of these standards is the same for both VA and non-VA healthcare entities and individuals. Moreover, the sociodemographic characteristics of the VA workforce are not markedly dissimilar to those of other U.S. healthcare facilities except with regard to the number of workers who served in the military. As noted, prior military service generally correlates with better household preparedness, suggesting that the preparedness of other non-VA, U.S. healthcare workers may be somewhat less as a consequence.

## Conclusions

Healthcare workers, including both clinical and non-clinical staff, play a critical role in disaster response when an event occurs. Accordingly, understanding their perceptions of their medical facility’s preparedness can help develop and implement more effective disaster policies and procedures, which in turn should ultimately lead to a better prepared, more resilient healthcare system for future crises. The findings from this study suggest both a desire and a need for additional trainings, particularly for epidemics. The outbreak of the novel coronavirus that causes COVID-19 underscores the need for training and other preparations for infectious diseases that was identified in our study.

Since non-clinicians were less likely than their clinical colleagues to perceive their role to be important during disaster response, training programs should also target non-clinical staff and describe the importance of their roles during disasters. This is particularly important as prior research suggests that perceptions of the importance of their role is a significant factor regarding whether healthcare workers report to work during disasters.

Additionally, communicating with employees regarding assurances of compliance and safety standards will increase confidence in their institution’s ability to respond. This could include the provision of information and training about the use of personal protective equipment, preventative medications or vaccines for staff and family, HEPA air purification and filtration units, or transportation options to and from work during a disaster.

## Supplementary information

**Additional file 1:.** VHA Preparedness Study Questionnaire.

## Data Availability

The datasets generated and/or analyzed during the current study are not publicly available since the data could potentially identify some of the respondents when linked to other data but are available from the corresponding author on reasonable request.

## Abbreviations

VA: U.S. Department of Veterans Affairs
VEMEC: Veterans Emergency Management Evaluation Center
