# Supplementary material for: Workforce preparedness for disasters: perceptions of clinical and non-clinical staff at the U.S. Department of Veterans Affairs
Source: BMC Public Health. 2020 Oct 2;20:1501. doi: 10.1186/s12889-020-09597-2 (PMC7531065; doi:10.1186/s12889-020-09597-2)
Supplement: Supplementary file 1 — Additional file 1:. VHA Preparedness Study Questionnaire. [file 12889_2020_9597_MOESM1_ESM.docx]

**VHA Preparedness Study Questionnaire**

The U.S. Department of Veterans Affairs, Veteran’s Health Administration (VHA), is conducting this survey to better understand how VHA employees prepare for and respond to disasters. Survey results will aid in a comprehensive assessment of VHA employees’ experience and perspectives regarding disaster preparedness. The results will also help guide program and policy development to benefit VHA preparedness. This survey will take approximately 15 minutes to complete. Participation in this survey is voluntary and your responses are confidential.


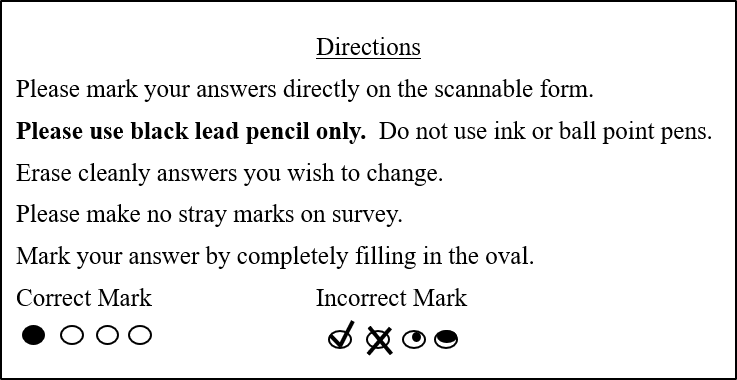


**PLEASE READ ALL INSTRUCTIONS BEFORE BEGINNING THE SURVEY.**

**SECTION A: DISASTER PREPAREDNESS AND PLANNING AT WORK**

The questions in this section ask about your preparedness and the preparedness of your VA Medical Center or VA Health Care System (VAMC/HCS) for major disasters in general, and three specific types of disasters. A major disaster is one that causes mass injury, loss of life, or widespread damage to property, and requires resources outside of the local community to help with recovery efforts.

|  | Yes | No | Don't Know |
| --- | --- | --- | --- |
| A.1 Have you ever been at the VA when a major disaster (not a drill) occurred? |  |  |  |
| A.2 Have you decided on a person (or persons) who would take care of your family obligations in case you are called into work during a major disaster? |  |  |  |

A.3 Read each statement below and indicate how strongly you agree or disagree. These statements refer to **any type of major disaster, natural or man-made**.

|  | Strongly Agree | Agree | Neither Agree or Disagree | Disagree | Strongly Disagree | Don’t Know |
| --- | --- | --- | --- | --- | --- | --- |
| a. My VAMC/HCS is prepared to respond to major disasters. |  |  |  |  |  |  |
| b. My knowledge about disaster preparedness is sufficient for carrying out my responsibilities at work during a major disaster. |  |  |  |  |  |  |
| c. My VAMC/HCS has encouraged employees to prepare their home and family for a major disaster. |  |  |  |  |  |  |
| d. I would like more information about preparedness for a major disaster. |  |  |  |  |  |  |

A.4 Now consider a **natural disaster**, such as an earthquake, hurricane, tornado, flood, wildfire, or severe winter storm.

|  | Strongly Agree | Agree | Neither Agree or Disagree | Disagree | Strongly Disagree | Don’t Know |
| --- | --- | --- | --- | --- | --- | --- |
| a. I am confident in my VAMC/HCS’s ability to respond to a natural disaster. |  |  |  |  |  |  |
| b. I would like additional training to prepare for a natural disaster. |  |  |  |  |  |  |
| c. I understand my role in my VAMC/HCS’s overall response to a natural disaster. |  |  |  |  |  |  |
| d. My role in VAMC/HCS’s overall response to a natural disaster is important. |  |  |  |  |  |  |

A.5 Now consider **an epidemic** (a widespread infectious disease), such as a pandemic influenza.

|  | Strongly Agree | Agree | Neither Agree or Disagree | Disagree | Strongly Disagree | Don’t Know |
| --- | --- | --- | --- | --- | --- | --- |
| a. I am confident in my VAMC/HCS’s ability to respond to an epidemic. |  |  |  |  |  |  |
| b. I would like additional training to prepare for an epidemic. |  |  |  |  |  |  |
| c. I understand my role in my VAMC/HCS’s overall response to an epidemic. |  |  |  |  |  |  |
| d. My role in VAMC/HCS’s overall response to an epidemic is important. |  |  |  |  |  |  |

A.6 Finally, consider a **radiological disaster**, such as a leak from a nuclear power plant or a dirty bomb.

|  | Strongly Agree | Agree | Neither Agree or Disagree | Disagree | Strongly Disagree | Don’t Know |
| --- | --- | --- | --- | --- | --- | --- |
| a. I am confident in my VAMC/HCS’s ability to respond to a radiological disaster. |  |  |  |  |  |  |
| b. I would like additional training to prepare for a radiological disaster. |  |  |  |  |  |  |
| c. I understand my role in my VAMC/HCS’s overall response to a radiological disaster. |  |  |  |  |  |  |
| d. My role in VAMC/HCS’s overall response to a radiological disaster is important. |  |  |  |  |  |  |

**SECTION B: DISASTER PREPAREDNESS & PLANNING AT HOME**

The next series of questions ask about steps you have taken to prepare yourself or your household for a major disaster.

B.1 How prepared do you feel your household is to handle a major disaster?

Well prepared

Somewhat prepared

Not prepared at all

|  | Yes | No | Don't Know |
| --- | --- | --- | --- |
| B.2 Do you have supplies set aside in your home to be used only in the case of a disaster? |  |  |  |
| B.3 Does your household have a 3-day supply of water for everyone who lives there? A 3-day supply of water is 1 gallon of water per person per day. |  |  |  |
| B.4 Does your household have a 3-day supply of nonperishable food for everyone who lives there? By nonperishable we mean food that does not require refrigeration or cooking. |  |  |  |
| B.5 Does your household have a working battery operated radio and working batteries for your use if the electricity is out? |  |  |  |
| B.6 Does your household have a working flashlight and working batteries for your use if the electricity is out? |  |  |  |
| B.7 Does your household have an emergency plan that includes instructions for household members about where to go and what to do in the event of a disaster? |  |  |  |

B.8 Does your household have a 3-day supply of prescription medication for each person who takes prescribed medicines?

Yes

No

Don’t Know

No one in the household requires prescribed medicines.

B.9 Do you have any pets, service animals, or large animals?

Yes

No (IF ‘NO’ GO TO B11)

B.10 Do you have any of the following for your pets, service animals, or large animals in the event of a major disaster?

|  | Yes | No |
| --- | --- | --- |
| a. Emergency supplies (food, water, litter). |  |  |
| b. Medications and medical/vaccination records. |  |  |
| c. Sturdy leashes, harnesses, and/or carriers to transport pet(s). |  |  |
| d. A list of locations that allow animals in an evacuation (workplace, shelters, boarding facilities, hotels, etc.). |  |  |

B.11 If public authorities announced a mandatory evacuation from your community due to a major disaster, would you evacuate?

Yes

No

Don’t know/not sure

B.12 Have you ever volunteered to help in a disaster?

Yes

No

B.13 Please read each statement below and indicate how strongly you agree or disagree.

|  | Strongly Agree | Agree | Neither Agree or Disagree | Disagree | Strongly Disagree |
| --- | --- | --- | --- | --- | --- |
| a. Getting information about how to prepare for a disaster is too hard. |  |  |  |  |  |
| b. I don’t know how to get prepared. |  |  |  |  |  |
| c. I don’t have time to prepare. |  |  |  |  |  |
| d. Preparing is too expensive. |  |  |  |  |  |
| e. I don’t want to think about preparing for disasters. |  |  |  |  |  |
| f. I have never thought about preparing for disasters. |  |  |  |  |  |
| g. If there were a disaster, the police and fire would take care of my needs. |  |  |  |  |  |
| h. I don’t need training to know how to react in a disaster. |  |  |  |  |  |
| i. My job encourages me to have a family disaster plan. |  |  |  |  |  |
| j. My job encourages or requires me to take training to prepare for disasters. |  |  |  |  |  |
| k. People I know have taken steps to get prepared. |  |  |  |  |  |
| l. Disasters in other places make me think about getting prepared. |  |  |  |  |  |
| m. Disasters I have experienced make me think about getting prepared. |  |  |  |  |  |

B.14 Have you personally experienced a disaster in the past? (Mark all that apply).

Yes, I was at home during the disaster

Yes, I was at work during the disaster

Yes, I traveled to respond to a disaster

Yes, Other

No

The next questions ask about disaster resilience.

B.15 Please read each statement below and indicate how strongly you agree or disagree.

|  | Strongly Agree | Agree | Neither Agree or Disagree | Disagree | Strongly Disagree |
| --- | --- | --- | --- | --- | --- |
| a. I look for creative ways to alter difficult situations. |  |  |  |  |  |
| b. Regardless of what happens to me, I believe I can control my reaction to it. |  |  |  |  |  |
| c. I believe I can grow in positive ways by dealing with difficult situations. |  |  |  |  |  |
| d. I actively look for ways to replace the losses I encounter in life. |  |  |  |  |  |

**C. GENERAL HEALTH AND HOUSEHOLD COMPOSITION**

C.1 In general, would you say your health is:

Excellent

Very Good

Good

Fair

Poor

C.2 Not including yourself, are there any people in your household in the following age groups? Mark ONE response for each category.

| Age Groups: | YES | NO |
| --- | --- | --- |
| 1. 0-5 years old |  |  |
| 1. 6-17 years old |  |  |
| 1. 18-64 years old |  |  |
| 1. 65 years and older |  |  |

C.3 Including yourself, does anyone in your household have a health condition that might affect your capacity to prepare for (respond to) to a disaster?

Yes

No

C.3.a. IF C3=YES 🡺 Do you have specific plans to meet your family’s health care needs in the event of a disaster?

Yes

No

C.4 Does anyone in your household need translation of English language instructions during a major disaster?

Yes

No

C.5 Are you or anyone in your household responsible for assisting someone who does not live with you?

Yes

No

C.6 Which of the following best describes the type of housing where you are currently living?

Single-family home

Apartment

Condominium or Townhome

Mobile home

Group housing (such as supportive, assistive, or transitional housing)

Other

**SECTION D: DEMOGRAPHICS**

The next questions are to help us better understand you and your household.

D.1 Have you ever served in the United States armed forces, either in the regular military or in a National Guard or military reserve unit?

Yes

No

D.2 What is your gender?

Male

Female

D.3 What is your age?

18-24 years

25-34 years

35-44 years

45-54 years

55-64 years

65 years or older

D.4 Are you Spanish, Hispanic, or Latino?

Yes

No

D.5 What is your race? (Mark all that apply)

White

Black or African American

American Indian or Alaskan Native

Asian

Native Hawaiian or Other Pacific Islander

Other

D.6 What is your marital status? (Mark ONE choice).

Now married

Currently living with partner, not married

Never married

Widowed

Divorced

Separated

D.7 What is the highest degree or level of school you have completed? (Mark ONE choice.)

Less than high school

High school diploma or equivalent (e.g., GED)

Vocational or technical school after high school

Some college or associate degree

Bachelor’s degree

Master’s, Professional or Doctorate degree

**SECTION E: VA EMPLOYMENT**

E.1 Which of the following best describes your current employment status at the VA? (Mark ONE answer.)

Employed full time

Employed part time

E.2 How long have you been with the VA?

Less than 1 year

1-3 years

4-5 years

6-10 years

More than 10 years

E.3 What is your level of supervisory responsibility?

None

Team Leader (informal; not responsible for performance ratings)

First Line Supervisor (formal; rates performance, e.g.: Foreman, Section Chief)

Manager (formal; rates performance e.g.: Division/Department/Service/Care Line managers)

Executive (formal; rates performance e.g.: Associate Director, Chief of Staff, Program Director, Nurse Executive)

Senior Executive (formal; rates performance e.g.: Network Director, Facility Director, Chief Medical Officer, Chief Officers, Deputy)

E.4 What type of setting do you spend at least 20% of your time in? (you may select up to 5 options)

Administrative (Non-Clinical)

Inpatient Care

Outpatient Care

Extended Care (e.g.: Community Living Center)

Research

Education

Affiliate

E.5 What is the main type of service you provide? (Mark ONE answer)

Administrative (Non-Clinical)

Dental

Emergency Medicine (Urgent Care, Emergency Department)

Facility Management Services

Home or Community Care

Imaging (Radiology, Nuclear Medicine)

Acute Care Inpatient

Intensive Care Unit- Critical Care

Laboratory and Pathology

Law Enforcement

Medical Specialty

Mental Health

Community Living Center

Pharmacy

Primary Care

Prosthetics or Sensory Aids

Rehabilitation Services

Research

Spinal Cord Injury

Surgery, Anesthesiology or Surgical Specialty Care

Other

Thank you for completing the survey.

For additional information on how to prepare for disasters, please visit: <https://www.ready.gov/>
